# Meaning in life among Norwegian outpatients with personality disorders: a cross-sectional study

**DOI:** 10.1186/s12888-025-07366-2

**Published:** 2025-10-16

**Authors:** Aryan Aghdami, Geir Pedersen, Elfrida Hartveit Kvarstein, Tatjana Schnell

**Affiliations:** 1 Østre Agder County Outpatient Psychiatric Unit, Sørlandet Hospital, Postbox 416, Grimstad, Norway; 2 Department for Research and Innovation, Division for Mental Health and Addiction, Oslo University Hospital, Postbox 4959, Oslo, Norway; 3 Institute of Clinical Medicine, University of Oslo, Postbox 1171, Oslo, Norway; 4 Existential Psychology Lab, MF School of Theology, Religion and Society, Postbox 5144, Oslo, Norway; 5 Humanistic University Berlin, Berlin, Germany

**Keywords:** Meaning in life, personality disorder, borderline personality disorder, avoidant personality disorder, dual personality disorder, psychosocial functioning, severity of personality disorder, dimensional model of personality disorders

## Abstract

**Background:**

Meaning in life (MIL) is associated with positive health outcomes, but is generally low among people suffering from borderline personality disorder (PD). Research has shown that MIL has a buffering effect on depression, as well as borderline traits such as suicidality and self-harm. However, to date, no studies have examined Meaning in Life (MIL) in relation to other prevalent personality disorders such as avoidant PD, nor have they investigated how PD severity influences MIL or whether MIL buffers the impact of PDs on psychosocial functioning.

**Methods:**

Norwegian outpatients (*N* = 1708) were assessed for PDs in specialized clinics, and measured for meaning in life, symptoms of depression, anxiety and impairment of psychosocial functioning. The data underwent correlational analyses, then grouped into sub-threshold PD, borderline PD, avoidant PD and dual PD (satisfying criteria for both) and tested for mean differences in MIL. Mean differences in MIL were explored across different levels of psychosocial functioning impairment. Finally, a moderation analysis tested whether MIL would buffer the effect that symptoms of depression had on impairment of psychosocial functioning.

**Results:**

As expected, correlational analyses showed a negative relationship between MIL and symptoms of ill mental health. A community sample demonstrated the highest MIL, followed by the sub-threshold PDs, the single PDs (either borderline or avoidant), and the Dual PDs with the lowest MIL. Impairment in psychosocial functioning showed significant differences between each level of impairment (low, moderate, severe), with varying results between the PD-groups across these levels. The moderation analysis only showed a buffering effect for the sub-threshold PDs, and not for any other group who received a diagnosis of PD.

**Conclusion:**

Having a PD is associated with a severe detriment to the level of MIL. There is no apparent difference in mean MIL between the two most prevalent *types* of PDs in healthcare (borderline and avoidant PDs). However, the current findings indicate that *severity* results in different levels of MIL, thus lending support to the dimensional perspective of personality disorder.

## Introduction

### Meaning in life and personality

Meaning in life (MIL) is a subjective experiential state including various meaning making processes culminating in evaluating the degree of meaning in one’s life [1]. There is a consensus among empirical meaning researchers that MIL is largely determined by a sense of coherence, purpose and significance [2, 3], while others also view belonging as a core feature of MIL [4–6]. Coherence refers to experiencing life as comprehensible and can involve identity and life narrative integration, as well as having a consistent worldview. Purpose is about unifying behavior with values in long-term goals, as well as having a sense of direction in life. Significance reflects the evaluation of one’s life as mattering or making a difference. Finally, belonging refers to a sense of interconnectedness, of having a place in this world.

Over the years, research has provided ample evidence that high MIL is associated with a wide range of health benefits, and that low MIL is associated with ill health. To mention a few specific examples, MIL has been shown to be associated with positive affect and well-being [7–9], better management of mortality awareness, death anxiety and terminal illness distress [10–13], good physical health, health promoting behavior and longevity [14–19], the facilitation of resilience towards adverse life experiences, adjustment and post-traumatic growth [20–24], as well as acting as a coping resource or having a buffering effect against stress, anxiety, depression, hopelessness and suicidality [25–31]. On the other hand, a lack of meaning in life was associated with a 1.9 times higher risk of mortality [32]. A crisis of meaning predicted suicidality [31], suicidal ideation [33] and suicide behavior [34], and it longitudinally exacerbated general mental distress [35]. Further studies found strong associations between crisis of meaning and symptom severity, dissociative and PTSD symptoms, neuroticism, anxiety, depression, general psychological distress, COVID-19 distress, and pessimism, as well as low hope, optimism, resilience and self-control [30, 34, 36–39].

Since it seems clear that having MIL is associated with better overall health outcomes, addressing the art of achieving MIL could be a matter of public health concern. Since Viktor Frankl’s [40] pioneering book about finding meaning in the face of adversity during the Holocaust, the existential burden to find, or create meaning has been conveyed as an individual responsibility. However, we live in a complex world of heterogeneous information, social structures, economic, political and environmental situations, which together with individual differences may affect the achievement of MIL. Achieving MIL in a concentration camp might have been possible for Frankl, but it is not necessarily the expected outcome.

Although some researchers have found that achieving MIL is relatively commonplace [41, 42], there *are* individual differences in MIL. Within the study of individual differences, personality, stable traits and dispositions across situations and lifespans are studied, as are lasting themes and motives in individuals’ life stories [43]. The literature indicates that there are differences in dispositional traits that affect achievement of MIL, as well as life circumstances and motives that affect the search for MIL [5, 44–46]. Some researchers have even proposed that the ability to achieve MIL is a personality trait or capacity itself [47, 48]. The psychiatry of personality – the medical field dedicated to understanding and treating disorders in personalities – adds yet another layer to how individual differences affect MIL. These individual differences are worth looking further into if it could be of help for more people to achieve MIL and hopefully the associated health and societal benefits.

### Personality disorders

Personality disorders (PDs) are mental disorders characterized by enduring problems within the domains of affectivity, impulse control, interpersonal functioning and cognition and are associated with impaired psychosocial functioning [49, 50]. The global prevalence of any PD is 7.8% across cultures [51]. PDs represent considerable personal burden and high societal costs [52, 53]. Up to half of psychiatric outpatients suffer from a PD [54], and often even a larger proportion in communal healthcare [55]. Due to high degrees of suffering, functional impairment, human resource demand and costs for society, continuous research and further development of treatment options are important.

Both diagnostic manuals of the International Classification of Diseases 11 [50] and the American Psychiatric Association’s Alternative DSM-5 model for personality disorders [49] suggests that coherence in identity and self-direction are among the core aspects of personality functioning which are disturbed among patients with PDs (2013, p. 762). Identity-coherence and self-direction (agency) overlap conceptually with the necessary conditions to achieve MIL according to meaning research, namely sense of coherence, goal orientation and significance.

Among PDs, a disproportionately large percentage of people with borderline personality disorders (BPD) and anxious-avoidant personality disorders (AvPD) seek medical attention [54]. Recent prevalence data among PDs in clinical settings in Norway show that 31% are diagnosed with BPD, and 39% with AvPD [56]. Studies of clinical samples also demonstrate high levels of PD comorbidity [57, 58]. In other words, it is commonplace for patients with BPD or AvPD to have some traits or fulfill the criteria for various other types of PDs as well, such as paranoid, obsessive-compulsive or narcissistic personality disorders, to name a few. One study showed that both the number of comorbid PD traits as well as baseline psychosocial impairment was related to severity of PD [56]. Patients who fulfill the criteria for both BPD and AvPD in particular, may suffer from adversity common to each respective diagnosis, but also a more severe condition with enhanced symptom distress and poorer psychosocial functioning. One of the aims of this paper is to assess such a synergistic burden.

BPD is marked by a core sensitivity and fear of being abandoned or rejected, associated with a highly unstable self-image, emotions, relations and impulsive behaviors [49]. Reckless behaviors that are indirectly or directly self-harming are common, along with suicidal behavior with up to 10% completing suicide, and for some, also chronic feelings of emptiness or transient dissociative or psychotic tendencies. Qualitative thematic analyses of life experience with BPD provide further insight into common struggles, revealing an unquenched longing for stability on a background of inner chaos containing intense emotional instability, impulsivity, lack of safety in relationships, unclear ideals, sense of worthlessness, anxiety, with little ability to self-comfort other than short term unhealthy dependencies [59].

AvPD revolves around a sensitivity and fear of criticism and ultimately humiliation and rejection [49]. These concerns are associated with feelings of inferiority and social inadequacy, leading to pervasive avoidance of social situations from study and work, to friendships and intimate relationships. A qualitative narrative review with thematic analyses of AvPD found some similar, and some dissimilar themes compared to BPD [60]. Both share an unquenched longing for connection, a sense of being overwhelmed by emotions, self-criticism and an unclear sense of self. However, the quality, experience and style differed quite markedly with BPD. While persons with BPD experience uncontrollable emotions and unstable sense of self, persons with AvPD experience a weak sense of self, with little emotions due to habitual ways of keeping them away. Furthermore, although common in lacking a stable connection with others, persons with AvPD fear and avoid opportunities to get close, and opt to subordinate to others and refrain from self-assertion, while persons with BPD report uncertainty surrounding the stability and mutuality in the relationships they invest in. The overarching conclusion from the review of AvPD was a profound *lack* of agency. The diagnostic and qualitative descriptions of BPD, on the other hand, point toward a chaotic or uncontrollable agency.

The direct relationship between MIL and BPD has received some attention by Marco and colleagues. They found that MIL was lower among patients with BPD compared to other serious mental disorders [61] and that three dimensions of MIL, namely coherence, purpose and significance, were negative predictors of BPD symptoms [62]. This means that the lower the score in these three dimensions underlying MIL, the higher the chance to observe symptoms of BPD. They also found that MIL acts as a buffer against the risk of suicide among BPD patients, and as predictive of less future non-suicidal self-harm [63, 64]. To the authors’ knowledge, the direct relationship between MIL and AvPD have not been similarly studied.

### MIL in relation to PD, other mental disorders and psychosocial functioning

PDs are commonly associated with comorbid suffering from depression and anxiety [65] as well as poor social functioning. The latter is demonstrated in different clinical and non-clinical samples, and include impairment of occupational, social, leisure and general functioning, regardless of other comorbid mental disorders such as major depression [66]. Generally, mental disorders are known to contribute to lower MIL [47]. Also, Steen, Berghuis and Braam [67] found that PD-patients with or without depression showed equal lack of meaning, purpose and direction in life, while non-PD out-patients had significantly more lack in meaning, purpose and direction if they were depressed compared to those who were not. Moreover, despite the presence of depression, they found that patients with PD revealed significantly more lack of meaning, purpose and direction in life than patients without PD. In a recent study, suffering from personality pathology and depressive symptomatology was shown to foster increased reflection about meaning [68]. Yet, studies have found that searching for meaning may be detrimental when MIL is low [69], and that mere reflection does not provide increased MIL [68]. Thus, while both poor social functioning and depressive or anxiety symptoms in themselves may lower MIL, the presence of PDs may additionally hinder and complicate the achievement of MIL, as well as exacerbate the suffering from low MIL if persons with PDs engage in searching and longing for MIL.

## Methods

### Aims

The overall aims of the current study were to investigate associations between MIL and commonly presenting PD conditions in clinical samples; BPD, AvPD, and dual BPD- AvPD. The following hypotheses were tested:

- H_1_. The overview of meaning research presented in the introductory section reveals that meaning in life (MIL) is normally correlated with better health in most respects. In line with this, the present study hypothesized that MIL would be negatively correlated with indicators of ill mental health, specifically anxiety, depression, and impairment of psychosocial functioning.
- H_2_. A detriment to the ability to sustain a coherent self-identity and lead a purposeful life is explicitly stated in diagnostic descriptions of PDs, further supported by clinical research indicating lower MIL. In accordance with the prevailing understanding of the relationship between meaning and PD, H_2_ in this study states that PDs are associated with significantly lower meaning compared to less severe or sub-threshold PDs among psychiatric outpatients, as well as the general community.
- H_3_. Poor psychosocial functioning is associated with ill mental health. PDs are also associated with poorer experience of meaning compared to other psychiatric groups. It is not certain to what extent the lowered MIL is affected by the severity of PD or impairment of psychosocial functioning itself. H_3_ states that the severity of PDs have an independent effect on MIL, beyond *mere* impairment of psychosocial functioning.
- H_4_. While H_2_ is concerned with the positive correlation between MIL and mental health, literature has also shown that MIL can act as a buffer against ill mental health, as a protective factor and resilience factor. In line with the general trends in research on meaning, H_4_ states that meaning does act as a moderator for the effect of depressive symptoms on psychosocial functioning impairment, regardless of PD-category.

### Design

The study used cross-sectional anonymous data from treatment units who were partakers of The Network for Personality disorders, a cross-regional multicenter research collaboration in Norway [70]. These units specialize in assessment and treatment of personality disorders. The dataset used in the current study comprises collected data from between 2017 and 2020.

### Sample

The sample was retrieved from the quality register from the Network [70], and was based on the initial assessment of patients before starting treatment.

Initially, the material comprised anonymous data from 2402 patients across 15 treatment units, wherein 1850 (77%) females. Mean age was 30 (SD = 9, range 17–67 years). Age is concentrated between 21 and 30, comprising 46% of the sample for males and 55% of the sample of females.

A sample reduction followed due to two main exclusion criteria. Firstly, patients with deferred diagnostic assessments were not taken into account, since they could not be grouped as either warranting a PD diagnosis or not. This rendered the study sample down to *n* = 1850. Secondly, due to missing data on the measure of MIL (See below), the final study sample comprised 1708 patients for all analyses with inferential statistics.

### Assessment

#### Evaluation of mental disorders

Clinicians within the Network conducted structured diagnostic interviews according to DSM-5 [49], using the Mini International Neuropsychiatric Interview [71], and the Structured Clinical Interview for DSM-5 Personality Disorders (SCID-5-PD) for assessment of PDs [72]. Clinicians were experienced and trained in use of diagnostic interviews for PDs and part of multidisciplinary teams also involving psychiatrists and specialized clinical psychologists. Evaluation followed the LEAD-procedure (Longitudinal, expert, all data; [73, 74]. For the purpose of the current study, patients who did not satisfy the formal criteria for a PD are considered sub-threshold PD. Patients who satisfy the criteria for either AvPD or BPD are considered AvPD *or* BPD, regardless of variable comorbidity with other PDs. As demonstrated in the introduction, such comorbidities are normal. Patients who satisfied the criteria for both AvPD *and* BPD are considered dual PD, regardless of comorbidities with other PDs, as this group has recently received attention for showing higher rates of ill mental health, as highlighted in the introduction.

#### Patient self-reports

All patients received the same battery of questionnaires administered before starting treatment and regularly throughout treatment. These are described in a former, recent Network publication based on the same time period [56]. The current study included only the initial assessment and the following measures described beneath.

#### Measures of depression anxiety, and psychosocial functioning

Depression levels were measured with the Patient Health Questionnaire 9 (PHQ-9; [75], with a Norwegian validated scale [76]. PHQ-9 includes nine items on a 0–3 scale, with scores above 9 indicating clinical depression levels. Anxiety levels were measured with the Generalized Anxiety Disorder 7 (GAD-7; [77], also with a Norwegian validated scale [78]. GAD-7 includes seven items on a 0–3 scale, with scores above 9 indicating clinical anxiety levels. The Work and Social Adjustment Scale (WSAS; [79]) measured psychosocial functioning, including five items on a 0–8 scale, reflecting occupational ability, private management, leisure management, social activity management and management of intimate relationships. For the current study, *level of psychosocial functioning* is operationalized by scores < 15 indicating mild impairment, scores between 15 and 30 indicating moderate to severe impairment and scores > 30 indicating extreme impairment, demonstrated as meaningful distinctions in a comparable sample and scale validation of WSAS for the Norwegian clinical population [80].

#### Markers of severity

In the current study, two indicators are considered as severity operationalizations of PD. The aforementioned level of psychosocial functioning is considered one marker of severity, wherein higher impairment indicates more severe personality dysfunction. The other considered marker is a gradient starting from sub-threshold PD, to single BPD or AvPD, to dual PD – each indicating a higher severity of personality dysfunction.

#### Measure of MIL

Severity Indices of Personality Problems was given to all patients in the sample (SIPP-118; [81, 82]). The SIPP-118 is a self-report questionnaire developed to measure core components of maladaptive personality functioning. One of its 16 facets measures *purposefulness*, within the factor of *identity integration*, and attempts to measure “The capacity to make life meaningful by creating the means as well as the opportunities for achievement and organizing time in life with one’s goals” [83] (p. 33). The seven items in the questionnaire that measure purposefulness contain both items directly capturing experience of meaning and items that correspond to goals and interests. As an example of the former, item 9 states “I am convinced life is worth living”, while an example of the latter, item 46 states “I try to live in the moment, because long-term goals are meaningless”. In the literature, purpose in life represents a sub-dimension of meaning in life alongside other dimensions such as sense of coherence and sense of significance [3, 5, 84].

We thus considered it necessary to avoid confounding a sense of purpose and the general experience of meaningfulness. Therefore, the items of the purposefulness facet were subjected to an exploratory factor analysis to determine their dimensionality. From this initial analysis cases with more than four of the total seven items missing were excluded from further analysis, hence the final sample size of *n* = 1708.

### Statistics

Four hypotheses were tested in this study, in addition to an exploratory factor analysis to gain a clear measure of experience of meaning, using IBM SPSS Statistics for Windows version 28.0 [85], including PROCESS for SPSS module version 4.0 for moderation analyses [86]. A priori power analyses were calculated with software G*power [87, 88].

First, we tested the SIPP-118 purposefulness scale’s dimensionality and reliability with a principal axis factor analysis (EFA), conducted on its seven items with direct oblimin rotation. An initial analysis was run to obtain eigenvalues for each factor in the data. The factors were then analyzed by factor loadings and inspected on a scree plot. A second principal axis factor analysis was then conducted requesting a one factor solution based on the analysis of the initial EFA. Finally, Cronbach’s alpha was calculated to estimate the scale reliability.

H_1_ was tested by analyzing the relationships between MIL, impairment of psychosocial functioning (WSAS), symptoms of depression (PHQ-9) and anxiety (GAD-7) with Pearson’s product moment correlation for all subjects. The covariates of gender and age were also explored during this stage. See Limitations for a note regarding gender. Interpretations of the magnitude of correlation coefficients are in accordance with Cohen [89], in that absolute coefficient of 0.10 are considered small, 0.30 are considered medium/moderate, and 0.50 are considered large.

For H_2_, a one-way ANOVA was conducted to test mean group differences in MIL across groups of sub-threshold PD, BPD, AvPD and Dual PD. Levene’s test of homogeneity of variances found that differences in variance were statistically significant, *F*(3, 1396) = 7.82, *p* <.001. Therefore, a Welch robust test of equality of means was also performed, as well as Games-Howell post-hoc tests. Unpaired T-tests of the purposefulness facet of SIPP-118 were performed between each clinical group (sub-threshold PD, BPD, AvPD and Dual PD) and community data from a separate study [81], for comparative purposes.

To test H_3_, differences between mean MIL scores were analyzed by conducting a two-way ANOVA to compare between PD-group differences in meaning across levels of low, moderate, and high functioning impairment (based on clinically tested cut-offs), with gender and age controlled for. A Levene’s test of homogeneity of variances for the current model showed significant results, *F*(11, 1386) = 2.77, *p* <.001, thereby violating the assumption of homogeneity of variances. Due to these violations, Welch robust tests of equality of means were performed. A Bonferroni-adjusted pairwise comparison of estimated marginal means for differences in MIL between each PD-group’s level of functioning with all levels of functioning was performed.

A moderation analysis was performed to test H_4_, namely whether higher meaning makes it easier to function better despite suffering depressive symptoms, with age and gender controlled for.

### Power analysis

The designs demanding highest power were the ANOVA and moderation analyses. Effect sizes were set to medium level. For the ANOVA, 12 groups represented *low functioning* sub-threshold PD, AvPD, BPD and dual PD, *moderate functioning* sub-threshold PD, AvPD, BPD and dual PD, as well as *high functioning* sub-threshold PD, AvPD, BPD and dual PD, with four covariates being functioning impairment, age, gender, and experience of meaning. The following input parameters were used for ANOVA [90]: *F* = 0.25, *alpha* = 0.05, power = 0.95, numerator df = 6, number of groups = 12, number of covariates = 4, revealed a required total sample size of *n* = 341. The sample of *N* = 1708 thus satisfies the minimally justifiable power standards (0.80) and is sufficient to reach a 95% power for a medium effect size, as well as for a small effect size (*F* = 0.1), requiring1369 participants to reach a 0.80 power.

The following input parameters were used for determining the needed sample size for linear multiple regression (moderation analyses): *F* = 0.15, *alpha* = 0.05, power = 0.95, total and tested predictors = 3, revealed a required total sample size of *n* = 119. The smallest group that was tested were the Dual PD (*n* = 130) which surpasses 119 participants.

## Results

### Factor analysis

The Kayser–Meyer–Olkin measure (KMO) of 0.77 verified sample adequacy for the analysis. The initial principal axis factor analysis of the purposefulness items of the SIPP-118 obtained two factors with eigenvalues above 1 accounting for 58% of the variance. The first factor accounted for 42% of the variance, and the second accounted for 16%. The factor with the second highest loading was at the point of inflection in the scree plot, indicating lower overall factor loadings and supporting a one-factor solution. The subsequent analysis extracted one factor. In accordance with Stevens [91] with respect to substantive factor loadings, all items with loadings > 0.4 were considered representative of this factor. They capture experiences of meaning and meaninglessness (items 9, 23, 35 and 60), whereas items with low loadings (< 0.4) on this factor tapped goals and interests (items 114, 46 and 72; see Table 1). The resulting MIL scale showed good reliability (α = 0.82) and was used as a measure of overall *experience of meaning* in the current study. The remaining items lacked sufficient consistency to be used as a separate measure of purpose.

**Table 1:** Overview of purposefulness items of SIPP-118 loadings on one factor

Item #	Question	Loading
60	I often feel that my life is meaningless*	0.82
23	I often do not see a reason to keep on living*	0.80
9	I am convinced life is worth living*	0.79
35	Most of the time I am capable of spending my days in a meaningful way*	0.48
114	One of my problems is that I am missing clear goals in my life	0.39
46	I try to live in the moment, because long-term goals are meaningless	0.32
72	My interests change continuously	0.21

Principal axis factoring. *= items retained as MIL-variable in this study

### Hypothesis 1: correlational analyses

MIL showed a moderate negative correlation with impairment of psychosocial functioning (*r* = –.45, *p* <.001), a strong negative correlation with depression (*r* = –.56, *p* <.001), a low-moderate negative correlation with symptoms of anxiety (*r* = –.28, *p* <.001), and a low positive correlation with age (*r* =.20, *p* <.001) (see Table 2 for descriptive statistics, and Table 3 for a correlation matrix). Impaired functioning showed a high positive correlation with symptoms of depression (*r* =.61, *p* <.001) and a moderate positive correlation with symptoms of anxiety (*r* =.41, *p* <.001). Symptoms of anxiety and depression showed a high positive correlation (*r* =.64, *p* <.001). All estimated correlations are two-tailed.

**Table 2:** Descriptive statistics

Variable	M	SD	*N*
Gender	1.78	0.42	1708
Age	30.04	9.00	1708
Psychosocial functioning impairment	23.32	8.00	1705
Depression symptoms	17.88	5.52	1705
Anxiety symptoms	13.10	4.70	1683
MIL	2.30	0.80	1705

Gender. 1 = male, 2 = female. *MIL* Meaning in life. Ranges: Age (17-67), Psychosocial. (0-40), Depression (0-27), Anxiety (0-21), MIL (1-4).

**Table 3:** Correlation matrix

		Gender	Age	MIL	Psychosocial functioning impairment	Depression symptoms
Age	*r*	-.11^**^				
	*95% CI*	-.16 – -.06				
	*Sig.*	< .001				
MIL	*r*	0	.20^**^			
	*95% CI*	-.04 – ,05	.16 – .25			
	*Sig.*	.90	< .001			
Psychosocial functioning impairment	*r*	-.03	.04	-.45^**^		
	*95% CI*	-.08 – .02	-.01 – .09	-.48 – -.41		
	*Sig.*	.21	.08	< .001		
Depression symptoms	*r*	.05^*^	-.09^**^	-.56^**^	.61^**^	
	*95% CI*	.00 – .10	-.13 – -.04	-.59 – -.52	.58 – .64	
	*Sig.*	.04	< .001	< .001	< .001	
Anxiety symptoms	*r*	.11^**^	-.08^**^	-.28^**^	.41^**^	.64^**^
	*95% CI*	.06 – .15	-.12 – -.03	-.32 – -.23	.37 – .46	.62 – .67
	*Sig.*	< .001	< .001	< .001	< .001	< .001

Two-tailed significance testing, with pairwise deletion. **. Correlation is significant at the 0.01 level (2-tailed). *. Correlation is significant at the 0.05 level (2-tailed). *CI* Confidence interval. CI estimation based on Fisher's r-to-z transformation. *MIL* Meaning in life

### Hypothesis 2: mean differences in MIL

**Fig. 1:**
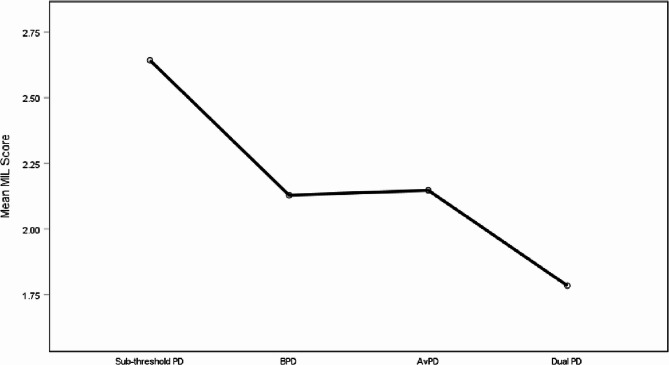
Mean MIL for respective PD-groups in sample. Note: MIL = meaning in life. Score range: 1 – 4. PD = personality disorder. Av = avoidant. B = borderline. Dual = patients with both avoidant and borderline personality disorders

A Levene’s test of homogeneity of variances found that differences in variance were statistically significant, *F*(3, 1396) = 7.82, *p* <.001. Therefore, a Welch robust test of equality of means was conducted and showed significant results, *F*(3, 523) = 53.36, *p* <.001), with a medium-large effect size, *η2* = 0.11 (*95% CI* = 0.078–0.137), *ω2* = 0.11 (*95% CI* = 0.076–0.135). Games-Howell post hoc tests showed significant differences among all groups at *p* <.001, except between the BPD and AvPD groups which were not significantly different (*Md* = − 0.019, *95% CI* = − 0.14–0.10, *SE* = 0.048, *p* =.979). Table 4 displays the descriptive statistics for MIL in the four groups. Table 5 and Fig. 1 display the mean differences among the groups, showing larger differences between the sub-threshold PD group with the highest scores in MIL compared to the PD groups, with the largest difference compared to the Dual PD group which had the lowest mean scores in MIL.

**Table 4:** Descriptive statistics for group differences in MIL

PD Group	*N*	Mean	SD	SE	95% CI for Mean	
Sub-threshold PD	352	2.64	0.83	0.04	2.56	2.73
BPD	417	2.13	0.71	0.03	2.06	2.20
AvPD	497	2.15	0.74	0.03	2.08	2.21
Dual PD	134	1.78	0.65	0.06	1.67	1.90
Total	1400	2.23	0.79	0.02	2.19	2.27

Scores range 1–4 on a likert scale. MIL = meaning in life. PD = personality disorder. B = borderline. Av = avoidant. Dual = patients with both borderline and avoidant PD

**Table 5:** Comparisons of MIL between PD-groups

PD Comparison		Md	SE	*p*	95% CI	
Sub-threshold PD	BPD	0.51**	0.06	< 0.001	0.37	0.66
	AvPD	0.50**	0.06	< 0.001	0.35	0.64
	Dual PD	0.86**	0.07	< 0.001	0.67	1.04
BPD	No PD	− 0.51**	0.06	< 0.001	− 0.66	− 0.37
	AvPD	− 0.02	0.05	0.98	− 0.14	0.10
	Dual PD	0.34**	0.07	< 0.001	0.17	0.52
AvPD	No PD	− 0.50**	0.06	< 0.001	− 0.64	− 0.35
	BPD	0.02	0.05	0.98	− 0.10	0.14
	Dual PD	0.36**	0.07	< 0.001	0.19	0.53
Dual PD	No PD	− 0.86**	0.07	< 0.001	−1.04	− 0.67
	BPD	− 0.34**	0.07	< 0.001	− 0.52	− 0.17
	AvPD	− 0.36**	0.07	< 0.001	− 0.53	− 0.19

Games-Howell Multiple Comparisons. Two-tailed significance test. **. Significant at the 0.01 level (2-tailed). *. Significant at the 0.05 level (2-tailed). *MD* mean difference; *MIL* Meaning in life; *PD* Personality disorder; *B* Borderline; *Av* Avoidant; *Dual* Patients with both borderline and avoidant PD

**Fig. 2:**
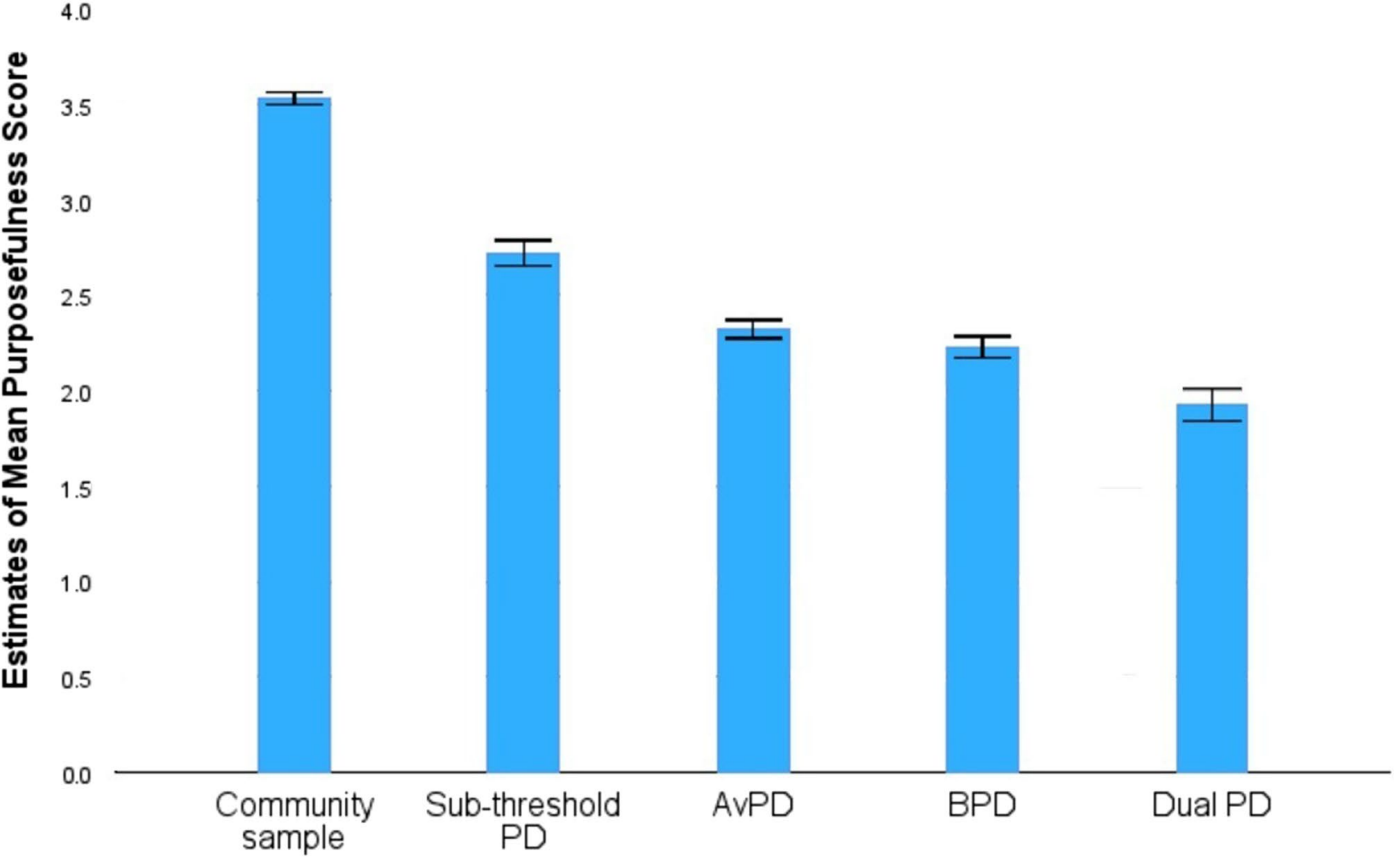
Mean levels of purposefulness in community sample compared with PDs. PD = personality disorder. Av = avoidant. B = borderline. Dual = patients with both avoidant and borderline personality disorder. Purposefulness is a facet within the factor *identity integration* within the Severity Indices of Personality Pathology scale from which the current MIL measure is derived (see Table 1). Error bars: 95% CI

For comparative reasons, T-tests including a community sample (*n* = 941) from a separate study [81] tested mean differences in purposefulness between the community sample and the respective groups of this study (see Fig. 2). The largest mean difference was between the Dual PD group and the community sample (*Md* = 1.61, *95% CI* = 1.52–1.70, *t*(672) = 35.13, *SE* difference = 0.05, *p* <.001). This was followed by the mean difference between BPD and the community sample (*Md* = 1.3, *95% CI* = 1.24–1.36, *t*(728) = 40.36, *SE* difference = 0.03, *p* <.001), and between AVPD and the community sample (*Md* = 1.21, *95% CI* = 1.15–1.27, *t*(752) = 41.12, *SE* difference = 0.03, *p* <.001). The lowest mean difference was found between the subjects with sub-threshold PD and the community sample (*Md* = 0.81, *95% CI* = 0.73–0.89, *t*(710) = 21.14, *SE* difference = 0.04, *p* <.001).

### Hypothesis 3: MIL per PD-group, across levels of impairment of psychosocial functioning

Welch robust tests of equality of means were performed across levels of functioning and found significant differences for sub-threshold PD, *F*(2, 130) = 29.58, *p* <.001, BPD, *F*(2, 122) = 23.88, *p* <.001, AvPD, *F*(2, 111) = 37.99, *p* <.001 and Dual PD *F*(2, 20) = 6.00, *p* =.009. Further Welch robust tests of equality of means were performed across PD-groups and found significant differences for severe functioning impairment, *F*(3, 134) = 7.11, *p* <.001, moderate functioning impairment, *F*(3, 305) = 20.49, *p* <.001, and low functioning impairment, *F*(3, 34) = 7.30, *p* <.001. The Welch analyses support robustness of the results despite violations of parameters for inferential statistics. See Table 6 for descriptive statistics.

**Table 6:** Descriptive statistics of MIL per level of functioning per PD-group

PD Group	Psychosocial functioning impairment	*N*	M	SD	SE	95% CI for M	
Sub-threshold PD	Low	89	3.14	0.75	0.08	2.98	3.29
	Moderate	208	2.56	0.77	0.05	2.45	2.66
	Severe	55	2.17	0.80	0.11	1.96	2.39
	Total	352	2.64	0.83	0.04	2.56	2.73
BPD	Low	51	2.58	0.79	0.11	2.36	2.80
	Moderate	258	2.18	0.67	0.04	2.09	2.26
	Severe	106	1.80	0.60	0.06	1.69	1.92
	Total	415	2.13	0.71	0.03	2.06	2.20
AvPD	Low	43	2.72	0.76	0.12	2.48	2.95
	Moderate	311	2.23	0.72	0.04	2.15	2.31
	Severe	143	1.79	0.60	0.05	1.70	1.89
	Total	497	2.15	0.74	0.03	2.08	2.21
Dual PD	Low	9	2.17	0.92	0.31	1.46	2.87
	Moderate	77	1.89	0.61	0.07	1.75	2.03
	Severe	48	1.54	0.59	0.08	1.37	1.71
	Total	134	1.78	0.65	0.06	1.67	1.90

*MIL* Meaning in life; *PD* Personality disorder; *B* Borderline; *Av* Avoidant; *Dual* Patients with both borderline and avoidant PD; *M* Mean MIL

The tests of between-subjects effects with MIL as dependent variable, showed significant main effects for age (*F*(1, 1384) = 70.34, *p* <.001, *ηp*^*2*^ = 0.05), PD-group (*F*(3, 1384) = 21.09, *p* <.001, *ηp*^*2*^ = 0.04), level of psychosocial functioning (*F*(2, 1384) = 66.40, *p* <.001, *ηp*^*2*^ = 0.09), but not for gender (*F*(1, 1384) = 1.57, *p* <.21, *ηp*^*2*^ = 0.0) or interaction effect (*F*(6, 1384) = 0.97, *p* <.44, *ηp*^*2*^ = 0.0). The explained variance was measured with Adjusted *R*^*2*^ = 0.249.

The comparison of MIL between each level of functioning impairment within each PD-group separately (sub-threshold PD, AvPD, BPD and Dual PD), showed significant differences in MIL between nearly all levels (see Table 7). One exception was for the dual PD sample, with no significant difference in MIL between the low and moderately impaired.

**Table 7:** Within group comparisons of MIL per level of psychosocial functioning

PD Group	Level of psychosocial functioning impairment		Md	SE	*p*	95% CI	
Sub-threshold PD	Low	Moderate	0.61**	0.09	< 0.001	0.41	0.82
		Severe	1.03**	0.12	< 0.001	0.75	1.31
	Moderate	Severe	0.41**	0.10	< 0.001	0.17	0.66
BPD	Low	Moderate	0.38**	0.11	< 0.001	0.13	0.63
		Severe	0.77**	0.12	< 0.001	0.49	1.05
	Moderate	Severe	0.39**	0.08	< 0.001	0.20	0.58
AvPD	Low	Moderate	0.49**	0.11	< 0.001	0.23	0.76
		Severe	0.99**	0.12	< 0.001	0.70	1.27
	Moderate	Severe	0.49**	0.07	< 0.001	0.33	0.66
Dual PD	Low	Moderate	0.29	0.24	0.67	− 0.29	0.87
		Severe	0.64*	0.25	0.03	0.05	1.24
	Moderate	Severe	0.35*	0.13	0.02	0.05	0.65

Bonferroni-adjusted comparisons based on estimated marginal means. **. Significant at the 0.01 level (2-tailed). *. Significant at the 0.05 level (2-tailed). *MD* Mean difference; *MIL* Meaning in life; *PD* Personality disorder; *B* Borderline; *Av* Avoidant; *Dual* Patients with both borderline and avoidant PD

**Fig. 3:**
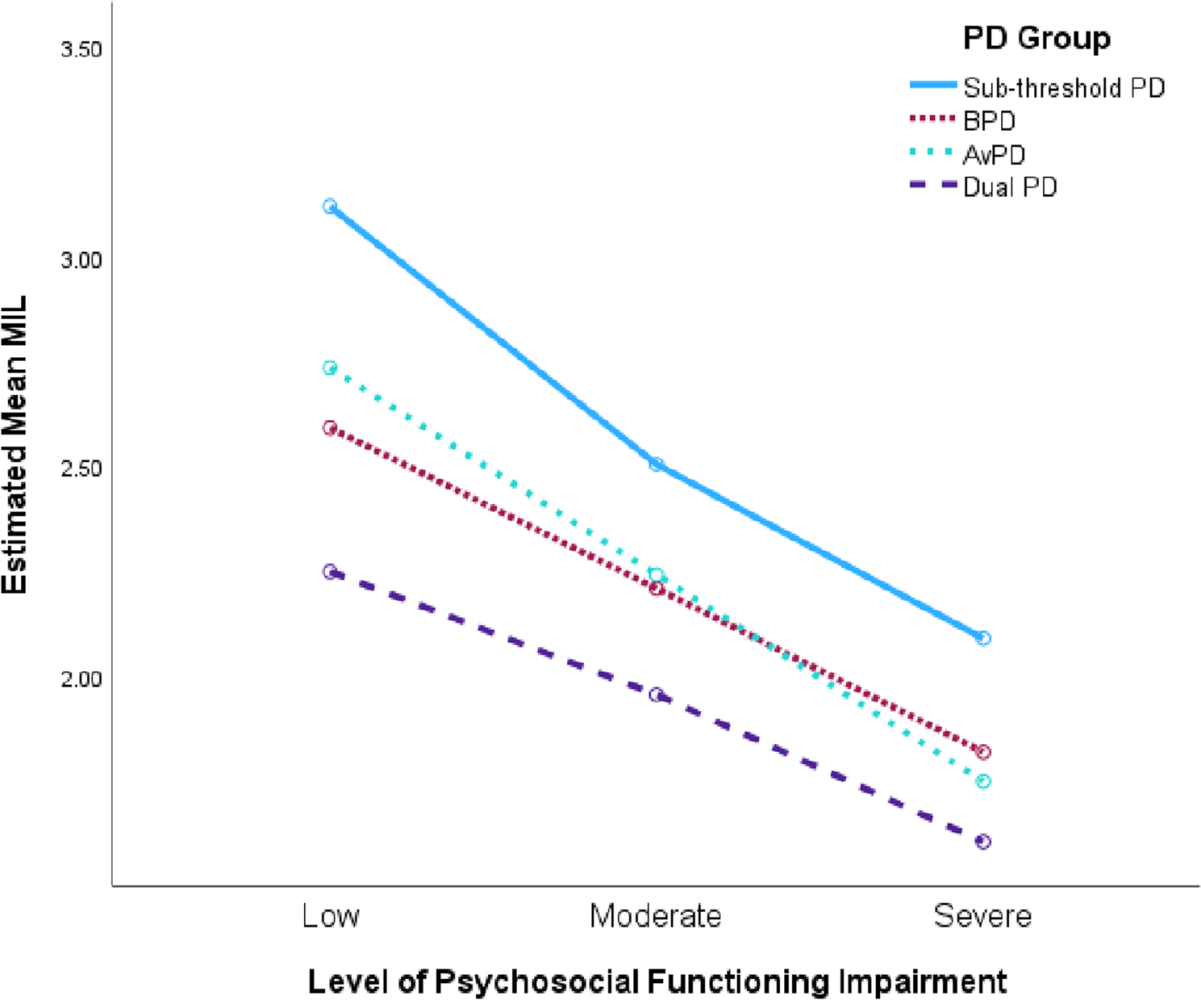
Mean MIL of PD-groups across levels of psychosocial functioning impairment. Note: PD = personality disorder. MIL = Meaning in life, based on estimated marginal means. Covariates evaluated at 1.7 for gender and 29.9 for age

The comparison of MIL within each level of functioning impairment, between all PD-groups, showed varying results (see Table 8). There were no significant differences between single PDs (AvPD compared to BPD) within any level of functioning impairment. At the low level of impairment, single PDs were not significantly different from dual PDs, but sub-threshold PDs were significantly different from all PDs, whether single or dual. At the moderate level of impairment, all groups differed significantly from each other, except between the two single PDs. At the high level of impairment, only the sub-threshold PDs differed significantly from the AvPD-group and Dual PD-group. The rest, between sub-threshold PDs and BPD, between the two single PDs, or between each single PD and Dual PDs, did not show significant differences. See Fig. 3 for a visual overview of the pattern of PD-groups per level of functioning.

**Table 8:** Between group comparisons of MIL per psychosocial functioning level

Level of psychosocial functioning impairment	PD Group		Md	SE	*P*	95% CI	
Low	Sub-threshold PD	BPD	0.53**	0.12	< 0.001	0.21	0.85
		AvPD	0.38*	0.13	0.02	0.05	0.72
		Dual PD	0.87**	0.24	< 0.001	0.24	1.50
	BPD	AvPD	0.14	0.14	1.00	− 0.52	0.23
		Dual PD	0.34	0.25	1.00	− 0.31	1.00
	AvPD	Dual PD	0.49	0.25	0.32	0.18	1.15
Moderate	Sub-threshold PD	BPD	0.30**	0.06	< 0.001	0.12	0.47
		AvPD	0.26**	0.06	< 0.001	0.10	0.43
		Dual PD	0.55**	0.09	< 0.001	0.30	0.79
	BPD	AvPD	0.03	0.06	1.00	− 0.19	0.12
		Dual PD	0.25*	0.09	0.03	0.02	0.49
	AvPD	Dual PD	0.29*	0.09	0.01	0.05	0.52
Severe	Sub-threshold PD	BPD	0.27	0.11	0.11	− 0.03	0.57
		AvPD	0.34*	0.11	0.01	0.05	0.63
		Dual PD	0.48**	0.14	< 0.001	0.12	0.85
	BPD	AvPD	0.07	0.09	1.00	− 0.16	0.30
		Dual PD	0.21	0.12	0.44	− 0.10	0.53
	AvPD	Dual PD	0.14	0.12	1.00	− 0.16	0.45

Bonferroni-adjusted comparisons based on estimated marginal means. ** = *p* <.001, * = *p* <.05. *MD* Mean difference; *MIL* Meaning in life; *PD* Personality disorder; *B* Borderline; *Av* Avoidant; *Dual* Patients with both borderline and avoidant PD

### Hypothesis 4: MIL as a moderator between depression and impairment of psychosocial functioning

The moderation analysis did not show any significant moderation effect for the total sample (*β* = 0.03, *SE* = 0.03, *t* = 0.98, *p* =.33, *95% CI* = − 0.03–0.10), collective PD groups (*β* = − 0.05, *SE =* 0.05, *t* = −1.02, *p* =.31, *95% CI* = − 0.14–0.04), for the BPD-group (*β* = − 0.06, *SE =.*07, *t* = − 0.80, *p* =.43, *95% CI* = − 0.20–0.09), for the AvPD-group (*β* = − 0.10, *SE =.*08, *t* = −1.23, *p* =.22, *95% CI* = − 0.27–0.06) or the Dual PD-group (*β* = 0.14, *SE =* 0.19, *t* = 0.73, *p* =.47, *95% CI* = − 0.24–0.52), *β*: unstandardized coefficients. However, a significant moderation effect was found for the sub-threshold PD-group (see Table 9).

**Table 9:** Coefficient table for MIL as moderator between depression and psychosocial functioning impairment for sub-threshold PDs

	β	SE β	T	*p*	95% CI	
Depression symptoms	0.75	0.08	9.68	< 0.001**	0.6	0.9
MIL	−1.92	0.57	−3.35	< 0.001**	−3.05	−0.79
Interaction	0.18	0.07	2.55	0.01*	0.04	0.31
Gender	−0.84	0.89	−0.94	0.35	−2.58	0.91
Age	0.19	0.04	5.15	< 0.001**	0.12	0.26

*R*^*2*^ = 0.45(5, 346). *SE* Davidson-MacKinnon heteroscedasticity-consistent standard errors (Hayes & Cai, 2007). **. Significant at the 0.01 level (2-tailed). *. Significant at the 0.05 level (2-tailed)

**Fig. 4:**
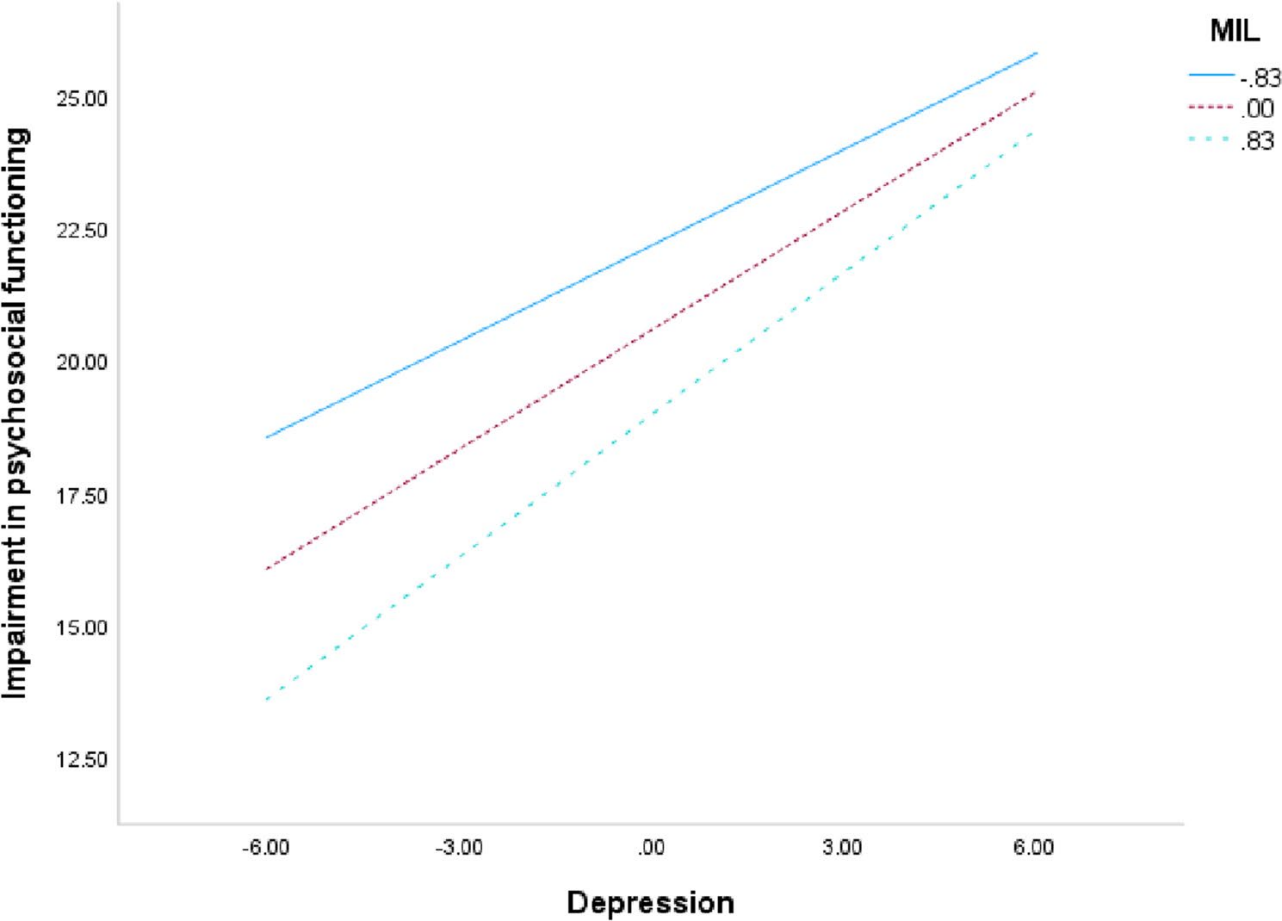
Moderation of MIL on the effect of depression scores on the impairment of psychosocial functioning. Note: MIL = Meaning in life. The figure depicts the moderation effect for the sub-threshold PD sample (*n* = 344). Centering by all variables that define product. Values conditioned at −1 SD, mean and + 1 SD. When MIL is lower (full line), any given depression score yields a higher functioning impairment, compared to depression scores when MIL is higher (double-dashed line). Covariates: gender and age

Table 9; Fig. 4 shows that when predicting a functioning impairment score by depression score, MIL moderates the prediction among sub-threshold PDs. In other words, when MIL is lower, any given depression score predicts a higher functioning impairment than the same depression score when MIL is higher. The moderating effect of MIL is strongest among lower depression scores.

## Discussion

The aim of this study was to explore the relationships between borderline (BPD), avoidant (AvPD) and dual personality disorders (Dual PD), and the experience of meaning in life (MIL) in a large Norwegian out-patient sample. The findings were in accordance with the previous literature. Our first three states hypotheses were confirmed while our fourth hypothesis, the moderation analysis was not. The results indicate the following: (1) The lower the MIL, the higher the scores in depression, anxiety and psychosocial impairment. (2) Severity of personality pathology was associated with lower MIL. Indeed, BPD and AvPD patients had significantly lower MIL compared to sub-threshold PD-patients, and Dual PD patients significantly lower MIL than BPD and AvPD respectively. (3) Personality pathology was associated with lowered levels of MIL beyond mere functional impairment. PD patients had lower MIL than sub-threshold PD patients, even when levels of functioning were comparable. (4) The association between depression and psychosocial impairment was not affected by MIL for patients with PDs. As a moderator, MIL only buffered the effect of depressive symptoms on psychosocial functioning for the sub-threshold patients and furthermore, the R² value indicated moderate overall variance explained by the model.

Past research has shed light on the lowered levels of MIL among BPD patients [61]. The current study is the first to demonstrate the equivalently lower levels of MIL among AvPD patients. The general dysfunctions for PDs include the inability to self-direct or form identity-coherence, according to the DSM-5 and ICD-11 [49, 50]. These dysfunctional abilities, corresponding with the concepts of purpose, significance and coherence, are also key components to achieve overall MIL [2, 3, 92]. However, even though the level of meaning was found to be equally low among BPD and AvPD patients, the pathways leading to the obstruction of MIL among AvPD and BPD could be different. One may speculate, for example, that while “reluctance to pursue goals” [49] (p. 765) may obstruct the possibility to achieve a direction in life among AvPD patients, “Instability in goals, aspirations, values, or career plans” (p. 766) may obstruct direction in life for BPD patients. Or, that while “unstable self-image” (p. 766) can make it difficult to achieve coherence in life among BPD patients, “self-appraisal as socially inept, personally unappealing, or inferior” (p. 765) could counteract a sense of significance and belonging among AvPD patients, each shown to be important for achieving overall MIL [2, 3, 5].

Respecting possibly different pathways to lowered MIL, despite equivalently lowered overall levels of MIL, may be relevant in the context of therapeutic measures. Successful interventions for BPD [93–96] have not been found to sufficiently cater the needs of patients with AvPD, requiring other approaches [97–102]. Thus, although both BPD and AvPD suffer from comparably low levels of MIL, different approaches may be necessary for each to enable meaning making and thus reaping the associated health benefits.

The current study is also the first to demonstrate that MIL was significantly lower for Dual PDs compared to patients with either BPD or AvPD. Comorbidities with other PDs are fairly common [57, 58]. Patients suffering from the combination of these two categories of PDs also demonstrated consistently lower psychosocial functioning compared to AvPD, BPD and sub-threshold PDs respectively, further indicating that the combination is associated with a higher severity of personality pathology. The findings are also in line with current state of the art research and revisions of personality disorder classification (ICD 11 and alternative model DSM 5), advancing a systematic evaluation of overall severity of personality problems in terms of both the quality and extent of dysfunctional areas.

More generally, this study found that the higher the level of psychosocial impairment, the lower the MIL across all groups. Yet, the overall trend showed that within each level of impairment, AvPD and BPD had lower MIL than sub-threshold PDs, and Dual PDs even lower than either AvPD or BPD. Our finding shows that there is no one-to-one relationship between level of psychosocial impairment and MIL. The only exception was for the dual PD sample, with no significant difference in MIL between the low and moderately impaired.

Personality pathology of increasing severity was generally associated with consecutively lower levels of MIL, within the same level of psychosocial impairment. One interpretation is that more severe personality pathology offers more obstruction to achieving dimensions of meaning such as coherence, purpose, significance, or belonging, resulting in less overall meaning, partly independent of the level of psychosocial dysfunction. Such an interpretation is in line with a salutogenic and health psychological perspective. Namely, that degree of adversity (e.g. impairment) does not itself constitute poor health, rather the lack of a sense of coherence, manageability and meaning in the face of adversity does [103]. “The sense of coherence reflects a person’s view of life and capacity to respond to stressful situations. It is a global orientation to view life as structured, manageable, and meaningful. It is a personal way of thinking, being, and acting, with an inner trust, which leads people to identify, benefit, use, and reuse the resources at their disposal ” (p. 67). Unfortunately, an essential feature of a PD seems precisely to be a global lack of such an inner sense of manageability and agency.

The interaction effect between PD group and level of psychosocial functioning was inconsistent with respect to subthreshold PDs. However, these subthreshold patients may represent a more heterogenous cohort, for clinical reasons referred to long-term treatment despite milder personality disturbance.

Contrary to our hypothesis based on previous findings that MIL acts as a buffer in various ways, the current study only found that MIL buffered the effect of depression on psychosocial impairment for the sub-threshold sample, and not for patients with AvPD and/or BPD. The lacking buffer effect may be interpreted in the context of previous research suggesting that psychosocial impairments among PDs are independent of co-occurring symptom disorders, such as major depressive disorder [66, 67]. Although the state of depressive symptoms has a clear negative correlation with MIL, the stable personality traits associated with PDs could be viewed as sustaining factors for psychosocial impairment and MIL. In other words, it might be more helpful to view the personality pathology and not depressive symptoms per se as the main obstruction for reaping the benefits of MIL as normally found in the unaffected population.

Although MIL has been found to buffer suicidality and future self-harm among BPD [63, 64], MIL does not seem to aid psychosocial functioning through buffering the state of depression. Taken together with previous findings suggesting that searching for meaning may be detrimental when the preconditions for achieving it are poor [68, 69, 104], alleviating personality pathology through means of specialized therapeutic approaches may be a precondition to obtain the psychosocial health benefits of MIL. However, research has also shown that therapeutic interventions focused on reducing pathology does not equate with improving skills with which to attain higher quality in life [105–107]. Thus, although therapeutic interventions for problematic personality traits may be a precondition to render the consequences of MIL more beneficial, including explicit focus on achieving MIL in therapies may still be an important addition, as has been suggested elsewhere [61, 108–111]. In this respect, healing and managing pathological personality traits may be viewed as a necessary condition for achieving MIL, but not necessarily a sufficient condition to achieve MIL, possibly requiring its own focus.

### Limitations

Data collection was performed in a clinical setting and based on regular clinical routines. Possible failures are likely to be randomly distributed and include a combination of failures in administration, software, local resources, therapists and patient factors. Investigation is described below, and did not reveal systematic missing-differences of note.

Of the 1850 patients with completed diagnostic assessment 145 patients had missing values on MIL. Comparing age and measures from PHQ-9, GAD-7 and WSAS between these and the 1705 patients with MIL no statistical differences were found (Independent samples T-test). A Chi-square test comparing these two groups on gender and the prevalence of the different PD diagnosis, including a PD diagnosis or not, two differences were disclosed. Among those in the non MIL group the prevalence of antisocial PD was 6% (*n* = 9), while among those with measures on MIL the prevalence was 2% (*p* <.01). Furthermore, among those without MIL the prevalence of avoidant PD was 24% (*n* = 35), while among those with measures on MIL the prevalence was 34% (*p* <.01). Although statistically significant we consider these differences as of no threat to the validity of our inferences or generalization of findings.

A simple correlation analysis reveals a Pearson r of 0.14 (*p* <.001) and Spearman’s rho of 0.18 (*p* <.001) between psychosocial functioning and diagnostic group. Furthermore a linear regression analysis, with depression as dependent- and psychosocial functioning and diagnostic group as independent variables revealed a Collinearity tolerance of 0.961 and VIF of 1.041. From this we find it reasonable to consider the inclusion of both psychosocial functioning and diagnostic group of no concern with respect to multicollinearity.

The majority of the sample were females (see Table 2). This is currently an expected gender disparity in clinical outpatient settings, whereby males are thought to have a harder time recognizing their own mental health issues and tend toward delaying help-seeking until symptoms reach a higher threshold of severity [112]. Unfortunately, there was no distinction between sex or gender during data collection for this study. The options were male or female, with no way to trace whether patients referenced their sex or gender. Though the sample is considered highly clinically representative, the generalizability of our findings across gender alternatives is uncertain.

A cross-sectional design does not allow inferences of causality. Nonetheless, the discussion part attempts to build on previous findings and clinical understanding, which include such inferences. For example, the current study found lower MIL among PDs, which was interpreted directionally. Previous research on MIL and PDs respectively, suggests that having a PD causes lower ability to achieve meaning, and not that lower meaning causes a PD.

In the current sample the bivariate correlation between the MIL factor of SIPP and WSAS is − 0.45 (*p* <.001). Thematically, these two operationalizations address very different subjective challenges. However, it is reasonable to suspect that the severity of despair reflected by the MIL factor is one of several possible clinical factors resulting in impairment of work and social functioning.

Second, because of the anonymization process, it was not possible to trace which patients came from which specialized unit. Unit-wise error could thus not be canceled out. However, since the data sample was so large with patients from across the whole country with equal assessment instructions among clinicians, systematic error from specific units are probably not of much concern for the generalizability of the current findings.

PDs are comorbid with many other psychiatric conditions, ranging from mood and anxiety disorders to post-traumatic stress disorder, eating disorders, neurodevelopmental disorders and learning disabilities. These disorders were not controlled for in the current study. The focal disorder in the current study was only the degree of PD, comparing patients with symptoms of personality disorder, either falling over or under the threshold for diagnosis during assessment.

The measure of severity of PD in this study can be considered less than optimal. Clinicians working with the Alternative Model for PDs and ICD-11 are currently testing and validating instruments to assess severity of PD [113, 114]. The current study did not have access to such alternative assessments and thereby had to utilize other means, such as measures of psychosocial functioning impairment or a sub-threshold PD, single PD, dual PD gradient, as *indicative* of severity.

The groupings for analyses did not control for specific combinations of PDs or amount of comorbid PDs, except for the combination of borderline and avoidant personality disorders. It means that the patients with single PDs (being categorized as BPD or AvPD) could have any other comorbid PD, any number of them, or none. A patient with BPD could also have paranoid personality disorder, or dependent personality disorder. Such comorbidities are common, and reflect an ecological validity in the variability among persons with PDs. Yet, based on clinical prevalence and preliminary research on the particular comorbid combination of BPD and AvPD, this group received particular focus. Following the rationale behind the dimensional model of PDs, detaching type of PD from severity of PD, any combination of comorbid PDs could in theory be mild, moderate or severe. With bigger data studies in the future, it should be possible to discern whether specific combinations of types or traits are associated with more severe outcomes.

Although the sample size was large and had good overall power for the analyses undertaken, the groups across functioning levels were unequal. For example, the number of high functioning Dual PDs were very low (*n* = 9) compared to other groups. The low sample size of this group might limit the generalizability of the findings. It may perhaps also be a demonstration of how detrimental this combination of PDs can be, thereby making it rare within high psychosocial functioning. Thus, its rarity could be viewed as support for considering dual diagnosis indicative of severity.

There were violations in the assumptions of homogeneity of variance and heteroscedasticity for the ANOVAs. This led to some measures taken into showing robustness of results by performing Welch tests and using Games-Howell comparisons.

### Future directions

Contemporary models for personality pathology have conceptualized poor self- and interpersonal functioning, overlapping with the capacities recognized as necessary for achieving MIL. One may argue that PDs provide very clear examples of the mental mechanisms that can limit MIL. Considering the dimensional aspect of PDs, further studying how meaning making is precluded by features of PDs may provide a lens to understand general hindrances in meaning making.

Meaning research has shown that some sources of meaning provide more meaning than other sources [115]. One future direction could be to include measures on sources of meaning pre- and post therapy, to understand general trends and variability among PDs, as well as possible changes in sources of meaning after successful therapy. In light of the schema therapeutic model of PDs, authenticity is lost due to living in survival modes, catering to imperatives of subjugation, self-sacrifice, unrealistic self-demands, approval seeking, and self-negligence [116]. Similarly, the mentalization based therapeutic model of PDs suggests that authenticity can be lost when engaging in non-mentalizing modes, such as e.g. *pretend mode*, involving a disconnection between inner and outer realities [117]. Thus, according to various therapeutic approaches to PDs, there is a high probability that personality pathology provides confusion on which potential sources of meaning are or could be authentic and not potential “pseudo sources”.

Finally, clinical research on existential and meaning-oriented therapies have found evidence for such approaches in therapy [118–120], but have not distinguished patients based on presence of personality pathology. Crises of meaning and existential anxieties may indicate states in which meaning-centered therapies could be beneficial. However, in light of the current study’s findings, problems in meaning making may be rooted in personality traits, as well. Therapists utilizing existential therapies may benefit from assessing aspects of personality pathology and consider applying treatment measures from personality therapies in parallel with their therapeutic approaches.

Considering the limitation of the cross-sectional design, further research on MIL should include a longitudinal design enabling assessment of change over time.

## Data Availability

No datasets were generated or analysed during the current study.
